# AcrIIA28 is a metalloprotein that specifically inhibits targeted-DNA loading to SpyCas9 by binding to the REC3 domain

**DOI:** 10.1093/nar/gkae357

**Published:** 2024-05-10

**Authors:** Gi Eob Kim, Hyun Ho Park

**Affiliations:** College of Pharmacy, Chung-Ang University, Seoul 06974, Republic of Korea; Department of Global Innovative Drugs, Graduate School of Chung-Ang University, Seoul 06974, Republic of Korea; College of Pharmacy, Chung-Ang University, Seoul 06974, Republic of Korea; Department of Global Innovative Drugs, Graduate School of Chung-Ang University, Seoul 06974, Republic of Korea

## Abstract

CRISPR–Cas systems serve as adaptive immune systems in bacteria and archaea, protecting against phages and other mobile genetic elements. However, phages and archaeal viruses have developed countermeasures, employing anti-CRISPR (Acr) proteins to counteract CRISPR–Cas systems. Despite the revolutionary impact of CRISPR–Cas systems on genome editing, concerns persist regarding potential off-target effects. Therefore, understanding the structural and molecular intricacies of diverse Acrs is crucial for elucidating the fundamental mechanisms governing CRISPR–Cas regulation. In this study, we present the structure of AcrIIA28 from *Streptococcus phage Javan 128* and analyze its structural and functional features to comprehend the mechanisms involved in its inhibition of Cas9. Our current study reveals that AcrIIA28 is a metalloprotein that contains Zn^2+^ and abolishes the cleavage activity of Cas9 only from *Streptococcus pyrogen* (SpyCas9) by directly interacting with the REC3 domain of SpyCas9. Furthermore, we demonstrate that the AcrIIA28 interaction prevents the target DNA from being loaded onto Cas9. These findings indicate the molecular mechanisms underlying AcrIIA28-mediated Cas9 inhibition and provide valuable insights into the ongoing evolutionary battle between bacteria and phages.

## Graphical Abstract

**Graphical Abstract:**
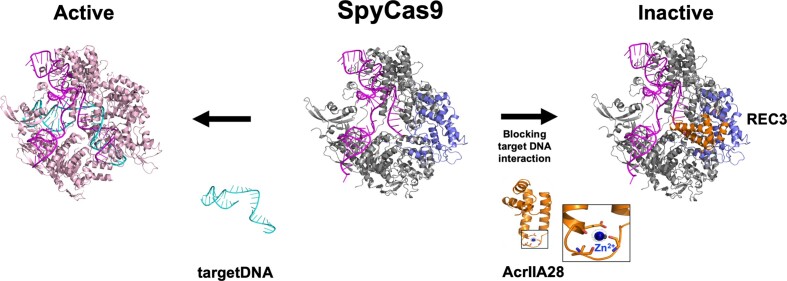

## Introduction

As a consequence of an extended evolutionary struggle for survival, bacteria have developed an intrinsic adaptive immune mechanism known as the clustered regularly interspaced short palindromic repeats (CRISPRs) and CRISPR-associated proteins (Cas) system (1,2). Within bacteria, the CRISPR–Cas system counteracts genetic material infiltration by phages by archiving recollections of previous infections in their CRISPR arrays (3). After subsequent infections, the CRISPR–Cas systems exhibit a prompt immune response through a two-phase process (3,4). During the expression phase, the CRISPR array harbored within the bacterial genome, containing processed and integrated segments of invading DNA, undergoes transcription and fragmentation, yielding small CRISPR RNAs (crRNAs) (5,6). Subsequently, the interference phase unfolds, wherein the crRNA pairs with Cas proteins to selectively identify specific DNA sequences from the invader, relying on the sequence information embedded in the crRNA for target recognition (7–10). Upon recognizing a complementary invader DNA sequence, the complex formed by the crRNA and Cas proteins catalyzes the cleavage of the invader's genetic material autonomously or by enlisting supplementary Cas proteins (7–10). Given the precision with which this bacterial immune system can incise specific DNA sites, CRISPR–Cas systems have found application in gene editing, enabling targeted modifications of genes for both experimental and medicinal objectives (11,12).

CRISPR–Cas systems are broadly categorized into two primary classes, Class 1 and Class 2, each encompassing six distinct types, denoted as type I–VI (9). These classifications are based on the composition of the CRISPR locus, the *Cas* genes present, and the underlying mechanisms (9,13). Class 1 CRISPR–Cas systems comprise types I, III and IV and consist of intricate multi-subunit RNA-guided surveillance complexes. In contrast, Class 2 CRISPR–Cas systems, including type II, V and VI, are characterized by the presence of a single protein effector molecule (13,14). Class 2 CRISPR–Cas systems, such as type II, feature a solitary effector protein called Cas9, which serves the dual function of target recognition and cleavage through interaction with a single guide RNA (15). For the type II CRISPR systems, Cas9 is adept at recognizing a protospacer adjacent motif (PAM), subsequently scanning neighboring DNA for sequences complementary to a segment located at the 3' terminus of the guide RNA, thus, establishing precise binding to its designated target (11,16). This interaction prompts a conformational alteration in Cas9 upon its binding to the target DNA, inducing an active state of DNA cleavage via a reconfiguration of the HNH and RuvC nuclease domains. Notably, these allosteric interactions play a pivotal role in modulating the cleavage activity of Cas9 (15,17–19). The inherent simplicity and accuracy of the CRISPR–Cas9 tool have propelled its widespread adoption as a prominent genome-engineering technique, particularly in gene editing, which can be utilized in both research and therapeutic applications (20–22).

In response to the relentless struggle for persistence, bacteriophages (phages) have developed a countermeasure in the form of anti-CRISPR proteins (Acrs) to thwart CRISPR–Cas systems, thus eluding bacterial immune defenses (23–25). Since the initial discovery of Acrs in 2013, about 100 Acr variants have been identified through functional screening and bioinformatic analysis (26). Given the frequent absence of sequence homology and common structural motifs among Acr proteins, their classification hinges on the specific CRISPR–Cas systems they target (25,27,28). For instance, Acrs that inhibit the type IF CRISPR–Cas system are designated AcrIF, whereas those targeting the type IIA CRISPR–Cas system are categorized within the AcrIIA family.

The AcrIIA family is known to inhibit the CRISPR–Cas9 system and has been identified in various bacterial species, suggesting it plays a widespread role in governing CRISPR–Cas activities. Previous investigations into AcrIIA9 and AcrIIA4 have yielded insights into the inhibitory mechanisms employed by this family, revealing direct interactions with Cas9 and Cas1–Cas2 complexes, respectively (29–31). While CRISPR-Cas systems have ushered in a new era of genome editing, providing a means for precise and efficient genetic manipulation (24,32), apprehensions surrounding potential off-target effects and unintended consequences necessitate a comprehensive exploration of their structural and molecular aspects. Thus, the intricate understanding of various Acrs is paramount for unraveling the nuanced mechanisms underlying the regulation of CRISPR–Cas systems. Moreover, despite identifying Acrs with potent inhibitory capabilities, the intricate details of how these Acrs inhibit Cas9 still need to be discovered.

AcrIIA28 is classified as an anti-CRISPR protein renowned for its capacity to impede the DNA cleavage activity inherent to the Cas9 enzyme (33). While the potent inhibitory ability of AcrIIA28 has been identified, the mechanisms by which it inhibits Cas9 have yet to be elucidated. In this study, we presented the determination of the 3D structure of AcrIIA28 from *Streptococcus phage Javan 128*. Moreover, we analyzed its structural and functional features to understand the molecular basis of the mechanism through which AcrIIA28 inhibits Cas9. We have demonstrated that AcrIIA28 is a metalloprotein containing Zn2+, and the presence of Zn^2+^ affects the activity of AcrIIA28. We found that AcrIIA28 specifically inhibits Cas9 from *Streptococcus pyrogen* (SpyCas9) but not Cas9 from other species by directly interacting with the REC3 domain of Cas9. Finally, we revealed that the direct interaction between AcrIIA28 and Cas9 prevents the target DNA from being loaded into Cas9, meaning Cas9 cannot cleave the DNA. These discoveries illuminate the molecular foundations of CRISPR–Cas inhibition orchestrated by AcrIIA28, offering valuable insights into the ongoing evolutionary battle between bacteria and phages. Moreover, a thorough comprehension of the structural and functional aspects of the AcrIIA family enhances our understanding of bacterial defense mechanisms and has the potential to unveil novel anti-CRISPR strategies.

## Materials and methods

### Cloning, overexpression, and purification of AcrIIA28, various Cas9 from different species and various Cas9 domains for structural and biochemical studies

Primer sequences used in this study are listed in Supplementary Table S1. The total length of the *AcrIIA28* (residues 1–88) gene in *Streptococcus Phage Javan128* (accession number: QBX23430.1) was synthesized by Bionics (Daejeon, Republic of Korea) and then cloned into a pET21a plasmid vector (Novagen, Madison, WI, USA), which contains a C-terminal hexa-histidine tag for affinity chromatography. The NdeI and XhoI restriction sites were used for cloning. After transforming the resulting recombinant construct into *Escherichia coli* BL21(DE3) competent cells, a single colony was selected and cultured in 1 l lysogeny broth at 37°C containing 50 μg/ml ampicillin until reaching a 600 nm optical density of 0.7–0.8. Then, 0.5 mM isopropyl-β-d-1-thiogalactopyranoside (IPTG) was added and incubated in a shaking incubator at 20°C for 18 h. The cultured cells were harvested by centrifugation at 2350 × *g* and 20°C for 15 min, resuspended in 20 ml lysis buffer (20 mM Tris–HCl pH 8.0 and 500 mM NaCl), and lysed via ultrasonication at 4°C. The cell lysate and the supernatant were separated by centrifugation at 27 000 × *g* and 4°C for 30 min. The separated supernatants were gathered, mixed with Ni-nitrilotriacetic acid (NTA) affinity resins, and incubated at 4°C for 3 h. Then, the incubated mixture was loaded onto a gravity-flow column (Bio-Rad, Hercules, CA, USA). After the mixture had flowed through, the resins were washed with an additional 50 ml lysis buffer to remove the impurities. Then, the resin-bound AcrIIA28 was eluted by adding elution buffer (20 mM Tris–HCl pH 8.0, 500 mM NaCl and 250 mM imidazole). The target protein samples were further purified by size exclusion chromatography (SEC) on a Superdex 200 Increase 10/300 GL column (GE Healthcare, Waukesha, WI, USA), previously equilibrated with SEC buffer (20 mM Tris–HCl pH 8.0 and 150 mM NaCl), and connected to an ÄKTA Explorer system (GE Healthcare). Eluted fractions were collected, pooled and concentrated to 19.5 mg/ml for structural and biochemical studies. The purity of the sample was visualized using sodium dodecyl sulfate-polyacrylamide gel electrophoresis (SDS-PAGE).

The *S. pyogenes* Cas9 (#62731), *N. meningitides* Cas9 (#71474), *H. parainfluenzae* Cas9 (#121540) and *S. muelleri* Cas9 (#121541) expression constructs were purchased from Addgene. The SpyREC12, SpyREC3, SpyHNH, SpyWED and SpyPI domain expression constructs were produced using cloning assays with purchased *S. pyogenes* Cas9 plasmids as templates. All the PCR products were cloned into the pET21a vector using NdeI and XhoI restriction sites. All the proteins were purified using the same method to purify AcrIIA28.

### Crystallization and X-ray diffraction data collection

Crystallization of AcrIIA28 was performed in an incubator at 20°C using the sitting drop vapor diffusion method. Initial crystals were obtained by mixing 1 μl of protein solution (19.5 mg/ml protein in SEC buffer) with 1 μl of reservoir solution (0.1 M HEPES pH 7.0, 0.5 M sodium phosphate, and 0.5 M potassium phosphate) and equilibrating the mixture using 0.5 ml of reservoir solution. The final crystals used for the X-ray diffraction study were grown on plates by equilibrating a mixture containing 1 μl of protein solution and 1 μl of reservoir solution containing 0.1 M HEPES pH 7.5, 0.8 M sodium citrate, 0.8 M potassium phosphate in two days. Subsequently, a single crystal was selected and soaked in a reservoir solution supplemented with 30% (v/v) glycerol for cryoprotection. X-ray diffraction data were collected at −178°C using the BL-5C beamline at the Pohang Accelerator Laboratory (Pohang, Korea). Data processing, which included indexing, integration, and scaling, was performed using HKL2000 software (34).

### Structure determination and refinement

The AcrIIA28 structure was determined using ARCIMBOLDO_BORGES, an *ab initio* phasing software (35), and by combining the fragment search using Phaser (36) and density modification using SHELXE (37). The initial model was generated automatically using AutoBuild from the Phenix package. Subsequent model building with refinement was performed using Coot (38) and phenix.refine (39). The structure quality and stereochemistry were validated using the MolProbity (40). All structural figures were generated using the PyMOL program (41).

### Multi-angle light scattering analysis (MALS)

SEC-coupled multi-angle light scattering (SEC-MALS) experiments were conducted to determine the absolute molecular mass of AcrIIA28 in solution. Purified AcrIIA28 protein in SEC buffer was injected into a Superdex 200 Increase 10/300 GL column pre-equilibrated with SEC buffer. SEC-MALS was performed at 20°C with a 0.5 ml/min buffer flow rate. The ÄKTA explorer system (GE Healthcare) was coupled with a DAWN-Treos MALS detector (Wyatt Technology, Santa Barbara, USA). A reference molecular mass value was established using bovine serum albumin for calibration. Data were processed and assessed using ASTRA software (Wyatt Technology).

### *In vitro* anti-CRISPR activity assay

To assess the inhibitory potential of AcrIIA28 and its mutants on Cas9-mediated target cleavage, a series of *in vitro* target DNA cleavage assays were conducted. SpyCas9 enzyme, acquired from NEB, was combined with a single guide RNA (sgRNA), generated using the HiScribeTM T7 Quick High Yield RNA Synthesis kit (NEB). Then, this mixture was incubated in NEBuffer r3.1 (NEB) at 20°C for 15 min. Subsequently, each wild-type AcrIIA28 and the mutant variants were introduced to the Cas9/sgRNA mixture and further incubated at 20°C for 15 min. Following the incubation step, target DNA was introduced into the reaction mixture, and the reaction was carried out at 37°C for 30 min. Proteinase K was added and incubated for 10 min to terminate the reaction. Subsequently, all reaction products were subjected to loading and separation through electrophoresis on 4% polyacrylamide gels. The visualization of DNA cleavage products was accomplished by staining the gels with SYBR GOLD. The resulting gel images were analyzed to evaluate the inhibitory impact of the wild-type AcrIIA28 and the various mutant forms on Cas9-mediated target cleavage.

### Inductively coupled plasma-mass spectrometry (ICP-MS) analysis

The concentration of trace metal ions in AcrIIA28 was determined through a comparative analysis involving a serial dilution of the Recipe® control samples (Munich, Germany), which were prepared in water. A combination of Be and Co internal standards was added to both the calibration points and the samples at specific concentrations to ensure accuracy. The measurements were performed using a NexION350D ICP-MS (Perkin-Elmer SCIEX model) and an Argon plasma source. This analysis was conducted at the National Center for Inter-University Facilities within Seoul National University (Seoul, Korea). The sample was introduced at a 1.00 ml/min rate during measurement. The obtained data presented in the results are the average values derived from triplicate samples, ensuring a robust evaluation of the trace metal ion concentrations in AcrIIA28.

### Size exclusion chromatography assay for complex formation

Size exclusion chromatography (SEC) was employed to investigate the formation of complexes between various Cas9s from different species and AcrIIA28, SpyCas9 domains and AcrIIA28, and SpyCas9 and multiple mutants of AcrIIA28. AcrIIA28 was mixed with various Cas9s and each Cas9 domain, followed by incubation at 4°C for 1 h. Numerous AcrIIA28 mutants were mixed with various Cas9s and each Cas9 domain and incubated at 4°C for 1 h. Then, the resulting mixture was loaded onto a Superdex 200 Increase 10/300 GL column (GE Healthcare) pre-equilibrated with SEC buffer (20 mM Tris–HCl pH 8.0, 150 mM NaCl). The eluted fractions corresponding to the peaks of interest were collected, and the samples were subsequently analyzed by SDS-PAGE stained with Coomassie Brilliant Blue. The migration patterns and the co-migration of protein bands within the gel were carefully examined and analyzed to assess the complex formation between AcrIIA28 or various mutants and the Cas9 proteins or individual domains.

### Native-PAGE

Protein–protein complex formation between SpyCas9 (or SpyREC3 domain) and AcrIIA28 was evaluated using native (non-denaturing) PAGE with 25% acrylamide gels. Coomassie Brilliant Blue was used to stain and detect the shifted bands. A concentration of 1 mM of each protein was used for this experiment.

### Isothermal titration calorimetry (ITC)

Nano ITC (TA Instruments) was used in the ITC experiments. For the ITC experiments, AcrIIA28 and SpyREC3 domain samples were prepared in a PBS buffer. Before titration, both protein samples were centrifuged at 14 000 rpm and 4°C for 10 min to remove debris. For titration, a concentrated AcrIIA28 (1.2 mM) was injected into a cell containing SpyREC3 domain (100 μM). All titrations were performed at 10°C with 25 injections performed at 200-s intervals. The binding isotherms were analyzed using the software provided by TA Instruments. Baseline controls were obtained from AcrIIA28 titrations to the buffer.

### Modelling of AcrIIA28/SpyCas9 complex structure

To generate a docking model of the AcrIIA28/SpyCas9 complex, the HDOCK server was utilized (42). The SpyCas9 structure used for the docking process was obtained from a previously solved structure within the Protein Data Bank (PDB) ID 5VW1, the target DNA-bound Cas9/sgRNA complex model (30). The default parameters provided by the HDOCK server were employed for the docking simulation.

### Mutagenesis

Site-directed mutagenesis was performed utilizing a QuickChange kit obtained from Stratagene (San Diego, CA, USA), following the manufacturer's instructions. Primer sequences used for mutagenesis are listed in Supplementary Table S1. The resulting mutations were subjected to sequencing analysis to verify the precision of the introduced alterations. This step ensured the fidelity of the modifications. Following successful validation, all mutant protein variants were produced and purified by applying the identical methodology previously described for the wild-type protein.

### Electrophoretic mobility shift assay with polyacrylamide gel (EMSA-P)

Purified target DNA (20 ng) was incubated with the AcrIIA28 + RNP complex or RNP alone in binding buffer (10 mM HEPES pH 7.5, 1 mM MgCl_2_ 20 mM KCl, 1 mM Tris-(2-carboxyethyl)-phosphine (TCEP) and 5% (v/v) glycerol, at a final volume of 20 μl) on ice for 30 min. Then, the prepared samples were separated by gel electrophoresis at 100 V on a 10% native 0.5 × TBE (Tris borate EDTA) polyacrylamide gel. AcrIIA28 was added to the RNP + DNA sample immediately prior to gel electrophoresis. After electrophoresis, gels were stained with SYBR Gold (Invitrogen, Waltham, MA, USA) and visualized according to the manufacturer's instructions.

## Results

### The high-resolution structural analysis of AcrIIA28 reveals a novel structure

**Figure 1.**
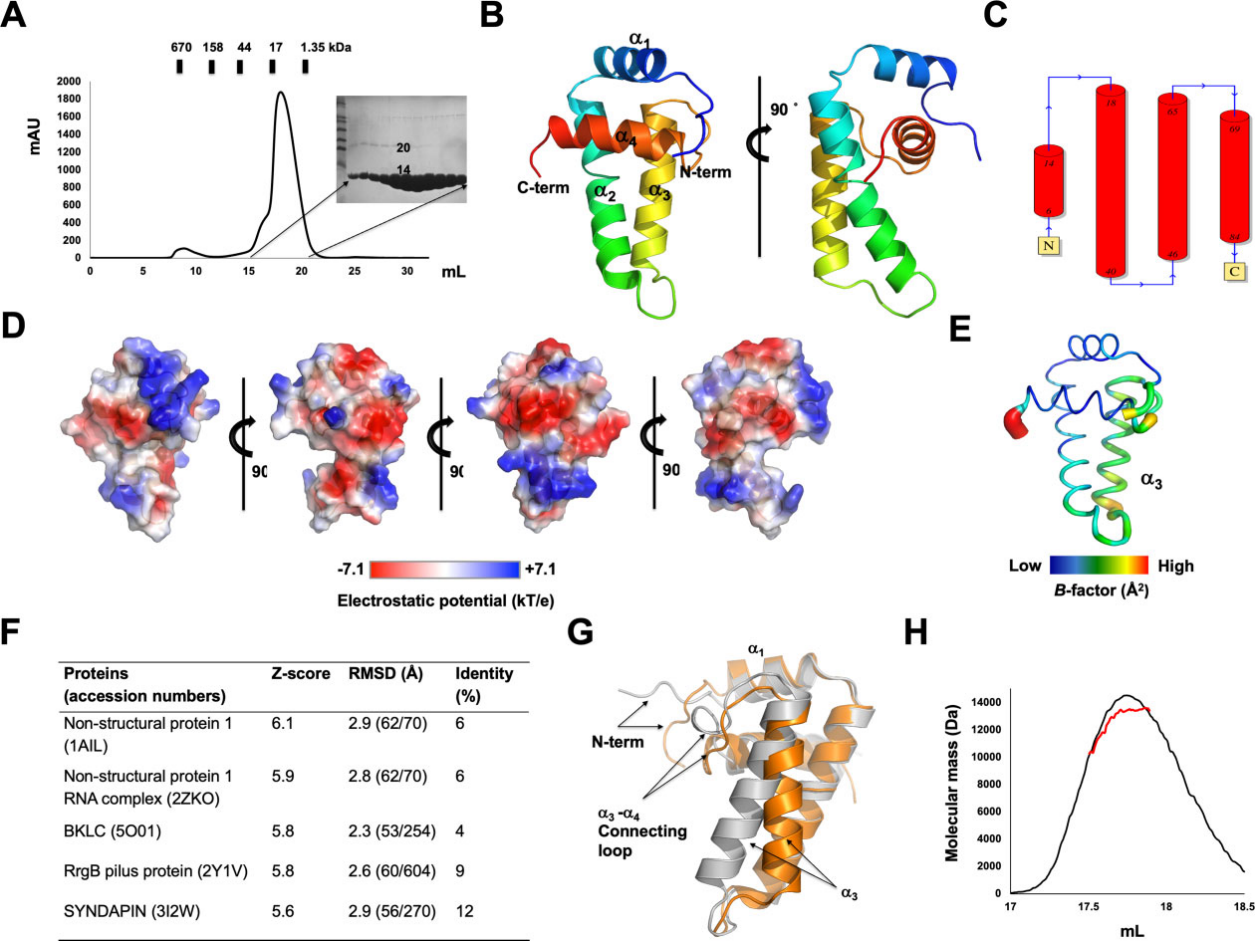
The high-resolution AcrIIA28 structure reveals a novel structural fold. (**A**) Size exclusion chromatography (SEC) profile of AcrIIA28. SDS-PAGE gel loaded with the main peak fractions is provided at the right side of the profile. Loaded fractions are indicated by the horizontal black bar. The corresponding fractions from SEC loaded onto SDS-PAGE are indicated by black arrows. (**B**) Cartoon representation of AcrIIA28 structure. The color of the chain from the N- to the C-termini gradually moves through the spectrum from blue to red. The four helices are labeled α_1_–α_4_. (**C**) Topology representation of AcrIIA28. (**D**) Surface electrostatic potential of AcrIIA28. The respective surface electrostatic distributions are represented by a scale ranging from −7.1 kT/e (red) to +7.1 kT/e (blue). (**E**) *B*-factor distribution in the structure of AcrIIA28. The structure is presented in a putty representation. Rainbow colors from red to violet with increasing *B-*factor values were used for *B-*factor visualization. (**F**) Table summarizing the result of structural similarity search using DALI server. (**G**) Structural superimposition of the experimental structure of AcrIIA28 (orange) with predicted structure by AlphaFold2 (grey). Structurally different regions that are not aligned are labeled. (**H**) SEC-multi-angle light scattering (MALS) profile of AcrIIA28. The experimental MALS data (red line) are plotted as SEC elution volume (x-axis) versus absolute molecular mass (y-axis) distributions on the SEC chromatogram (black) at 280 nm.

The investigation into the inhibition mechanism of AcrIIA28 was launched by performing the overexpression and purification of full-length AcrIIA28 for its subsequent structural analysis. The purification process encompassed Ni-NTA affinity chromatography followed by size exclusion chromatography (SEC). A distinct peak was observed throughout the SEC. SDS-PAGE analysis confirmed the purity of the AcrIIA28 protein in the main peak (Figure 1A). Attempted crystallizations were performed using protein samples eluted from the main peak, and successful crystallization was achieved. The diffraction data from a single crystal were collected at the PAL synchrotron.

The initial molecular replacement (MR) phasing method, carried out by employing a predicted structural model from Alphafold2 (43), failed. However, the *ab initio* phasing method using ARCIMBOLDO_BORGES software, which analyzes data on small helix and sheet fragments available in the PDB, was successfully conducted to solve the phasing issue. Finally, the 1.45 Å high-resolution structure of AcrIIA28 was determined, and the final structural model was refined to *R*_work_ = 19.75% and *R*_free_= 20.36%. The diffraction data and refinement statistics are summarized in Table 1. The crystal belonged to space group *P*4_1_2_1_2 and contained a single molecule in the asymmetric unit (ASU). The final structural model covered most of AcrIIA28 (residues M1 to Y86), excluding the last two C-terminal amino acids due to unclear electron density. The structure of AcrIIA28 consisted of four α-helices (α1–α4) (Figure 1B and C). Two major long helices in the centre (α2 and α3) is covered by two short helices from N- and C-terminal parts (α1 and α4) (Figure 1B). Analysis of the electrostatic potential on the surface indicated that both negative and positive charges were distributed equally to the surface of AcrIIA28 (Figure 1D). *B*-factor analysis highlighted high rigidity, with low *B*-factor values (average 25.3 Å) for most of the structure, except the α3 region (average 48.2 Å) (Figure 1E), underscoring the rigid conformation of the AcrIIA28 protein in solution.

**Table 1.** Data collection and refinement statistics

Data collection	
Space group	*P 4_1_ 2_1_ 2*
Unit cell parameter *a*, *b*, *c* (Å)	
*a*, *b*, *c* (Å)	*a* = 64.43, *b* = 64.43, *c* = 62.82
*α*, *β*, *γ* (°)	*α* = 90, *β* = 90, *γ* = 90
Resolution range (Å)	28.81–1.45
Total reflections	616 092
Unique reflections	24 054
Multiplicity^a^	25.6 (25.3)
Completeness (%)^a^	99.98 (100.00)
Mean *I*/σ(*I*)^a^	28.29 (2.41)
*R*_merge_^a,b^	0.06604 (1.474)
Wilson *B*-factor (Å^2^)	20.90
Refinement	
Resolution range (Å)	28.81–1.45
Reflections	24052
*R*_work_ (%)^a^	20.91 (27.03)
*R*_free_ (%)^a^	21.89 (25.83)
No. of molecules in the asymmetric unit	1
No. of non-hydrogen atoms	821
Macromolecules	733
Ligands	1
Solvents	87
Average *B*-factor values (Å^2^)	23.55
Macromolecules	22.41
Ligands	26.92
Solvents	33.14
Ramachandran plot:	
Favored/allowed/outliers (%)	100.00/0.00/0.00
Rotamer outliers (%)	0.00
Clash score	0.68
RMSD bonds (Å)/angles (°)	0.014/2.02

^a^Values for the outermost resolution shell in parentheses.

^b^ *R*_merge_ =Σ_*h*_ Σ_*i*_ |*I*(*h*)_*i*_ − <*I*(*h*)>|/Σ*_h_* Σ*_i_I*(*h*)*_i_*, where *I*(*h*) is the observed intensity of reflection *h*, and < *I*(*h*)> is the average intensity obtained from multiple measurements.

Exploring structurally similar proteins to AcrIIA28 using the DALI server (44) revealed that non-structural protein 1 (NS1), that is a protein from influenza virus, was the most structurally similar (Figure 1F). While NS1 emerged as the closest related structure, the low *Z*-score (6.1) and sequence identity (6%) indicated insufficient structural similarity. In addition, the other selected proteins also exhibited low *Z*-scores (around 5.9–5.6) and sequence identity (2–12%), indicating that the AcrIIA28 structure is novel, whose fold has not been reported previously.

Since the MR phasing method, which used the predicted structural model by Alphafold2, failed, we were curious to observe whether the predicted model was similar to the experimental structure. To accomplish this, we aligned two structures. Structural alignment of AcrIIA28 and the Alphafold2 predicted model revealed that the structures differed. Although the overall fold was the same, it was composed of four helices (Figure 1G). Notably, the locations of the N-terminal loop, α1 helix, α3 helix and the α3–α4 connecting loop were different from each other, although the starting and ending points of the four helixes were well matched (Figure 1G). This structural discrepancy caused the unsuccessful result for the MR phasing.

Indeed, while numerous Acrs are known to inhibit the activity of Cas9 as monomers, it has been demonstrated previously that certain Acrs exert their function in a dimeric configuration (12,45). In the case of AcrIIA28, the SEC result showed that AcrIIA28 was eluted between the molecular sizes of 17 and 1.35 kDa by producing one prominent peak in the chromatogram (Figure 1A). Since the SEC result was not sufficient to conclude the stoichiometry issue, to ascertain the stoichiometry of AcrIIA28, multi-angle light scattering (MALS) was employed. MALS analysis yielded an experimental molecular weight of 12.51 kDa (with a fitting error of 8.4%) (Figure 1H). Considering the theoretically calculated molecular weight of monomeric AcrIIA28 with a C-terminal 6X histidine tag, 11.48 kDa, the MALS data indicated that AcrIIA28 exists as a monomer in solution.

### AcrIIA28 effectively suppresses the DNA cleavage activity of Cas9, irrespective of the order of the reaction

**Figure 2.**
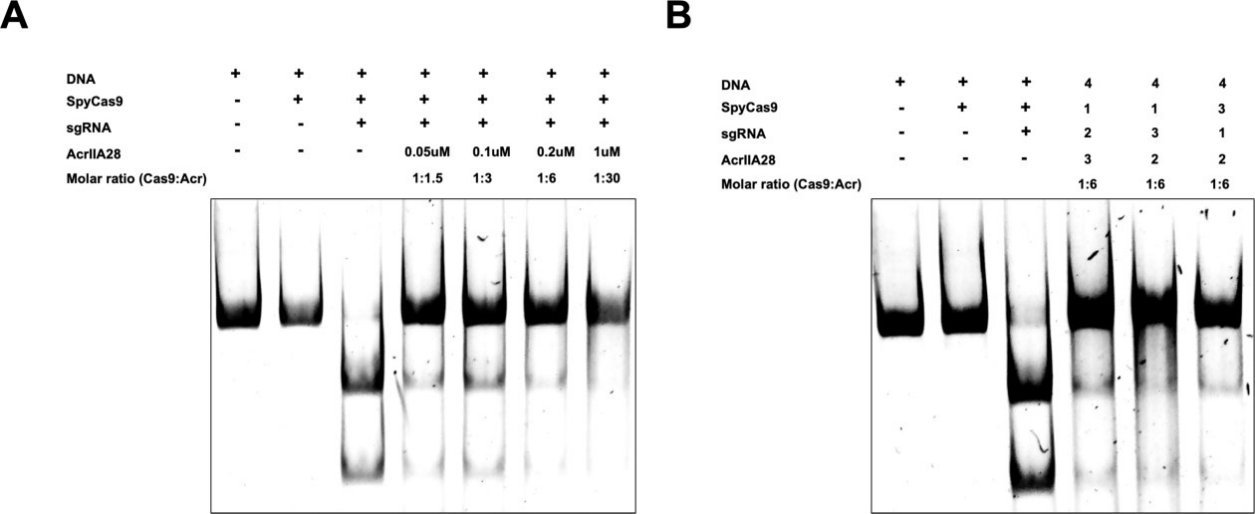
AcrIIA28 inhibits the target DNA cleavage activity of SpyCas9, regardless of reaction order. (**A**) An *in vitro* anti-CRISPR activity assay was used to detect Acr concentration-dependent inhibition of SpyCas9 activity. Polyacrylamide gels (4%) were stained with SYBR GOLD. The amount of AcrIIA28 added to the reaction is indicated. The numbers next to the agents used in the experiment indicate the order in which agents were added to the reaction: one represents the sample added first, and four represents the sample added last. In the enzyme reaction, + and – indicate added and not added, respectively. (**B**) An *in vitro* anti-CRISPR activity assay was performed to detect the reaction order-dependent inhibition of SpyCas9 activity. The numbers indicate the order in which the samples were added to the enzyme reaction. The experiments were performed three times with similar results.

To assess the inhibitory impact of AcrIIA28 on the enzymatic activity of Cas9, an *in vitro* target DNA cleavage assay was established. For this purpose, Cas9 from *Streptococcus pyrogens* (SpyCas9) was representatively selected for AcrIIA28’s inhibitory activity assay. SpyCas9, the most commonly employed enzyme in genome-editing applications, is a substantial nuclease consisting of 1368 amino acid residues. The assay used an 1100 bp double-stranded DNA (dsDNA) substrate containing the target region with the PAM sequence (5′-NGG-3′) of SpyCas9, along with a designed single guide RNA (sgRNA) for guiding SpyCas9 to the specific target site. The assay involved varying concentrations of AcrIIA28 to assess its inhibitory potential quantitatively. As anticipated, when SpyCas9 was incubated with sgRNA to form the ribonucleoprotein (RNP) complex, the target DNA was cleaved by SpyCas9 (Figure 2A). However, the addition of AcrIIA28 led to a gradual reduction in the cleavage of the target DNA with increasing concentrations of AcrIIA28 (Figure 2A). At an AcrIIA28 concentration of 1 μM, the cleavage activity of SpyCas9 was nearly completely inhibited. The band corresponding to the cleaved DNA fragment disappeared on the gel. Indeed, a low AcrIIA28 concentration of around 0.05 μM also effectively blocked the cleavage activity of SpyCas9, indicating that AcrIIA28 is an efficient and potent SpyCas9 inhibitor.

To further dissect the significance of the reaction order, additional assays were conducted with variations in the incubating reaction components. Despite the alterations in incubation order for each sample, the results consistently demonstrated robust inhibitory activity in all cases (Figure 2B). These supplementary assays indicate that the inhibitory impact of AcrIIA28 on SpyCas9 is not reliant on the reaction order.

### AcrIIA28 is a metalloprotein containing Zn^2+^ that affect the inhibitory activity of AcrIIA28 against Cas9

**Figure 3.**
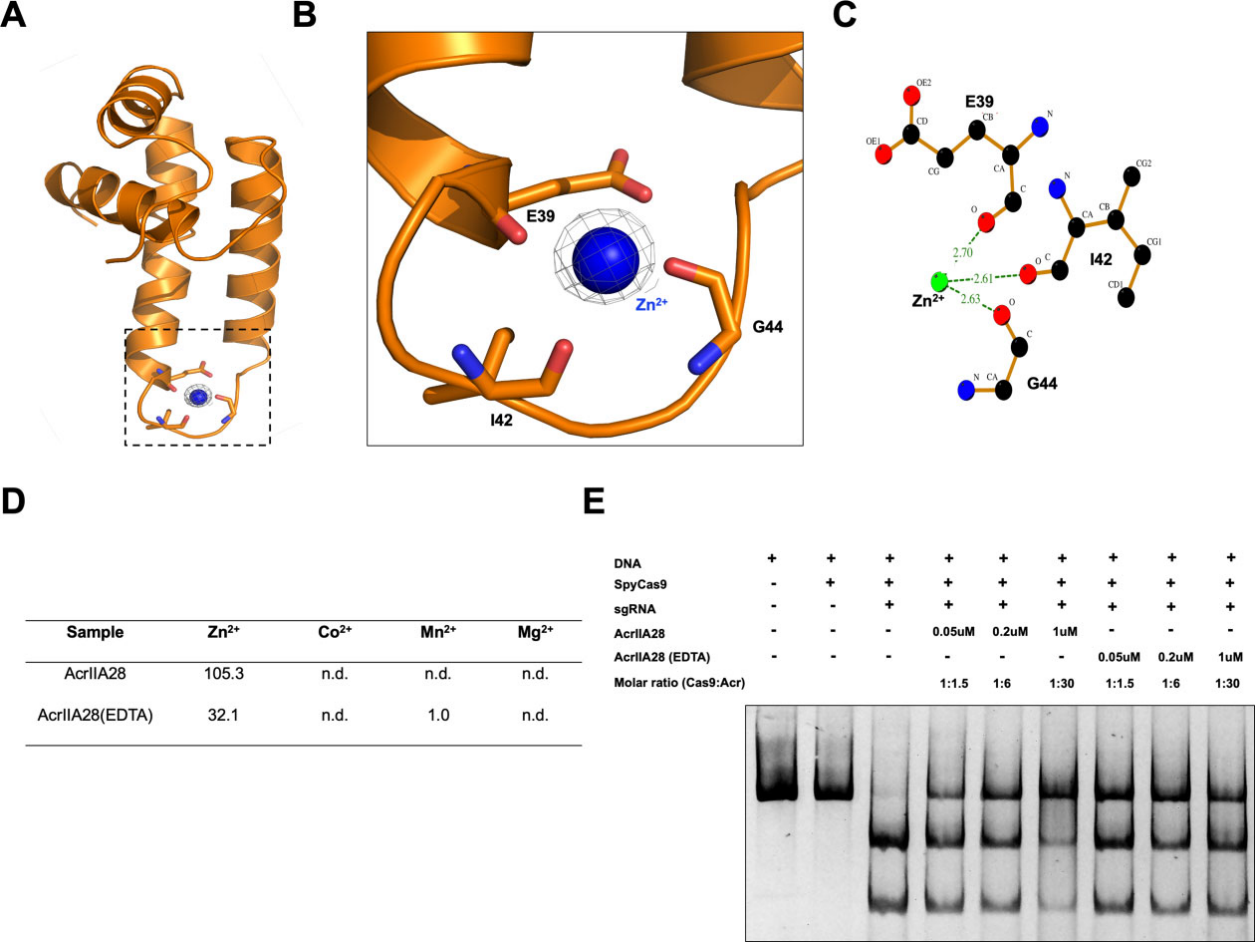
AcrIIA28 is a metalloprotein that coordinates Zn^2+^. (**A**) The electron density map around the metal ion binding site of AcrIIA28. The 2Fo-Fc map contoured at the 1σ level is shown. The magnified region displayed in panel (B) is indicated by a black-dot square. (**B**) Magnified cartoon of the metal binding site of AcrIIA28. Residues that coordinate with the metal ion are labeled. (**C**) LigPlot figure showing the environment of the metal binding site. The distances between the metal and each coordinated oxygen are indicated. (**D**) Table showing the metal ion concentration in AcrIIA28 (μg/kg) analyzed by ICP-MS. n.d.: not determined. (**E**) An *in vitro* anti-CRISPR activity assay of Zn^2+^ removed the sample of AcrIIA28 (EDTA). The numbers indicate the order in which the samples were added to the enzyme reaction: one represents the sample added first, and four represents the sample added last. In the enzyme reaction, + and – indicate added and not added, respectively.

During the structural refinement step, we found a big round-shape electron density, which exhibited a typical metal ion density, around the α3–α4 connecting loop region of AcrIIA28 (Figure 3A). This metal ion density was coordinated by oxygens from the main chain of E39, I42 and G44 (Figure 3B). The distance between metal ion and coordinated primary chain oxygen was less then 3 Å (Figure 3C). To reveal the identity of this metal ion, we used inductively coupled plasma mass spectrometry (ICP-MS). This analysis indicated that Zn^2+^ was the metal ion, with a detected concentration of 105.3 ppb (μg/kg) (Figure 3D).

After identifying that AcrIIA28 is a Zn^2+^ binding protein, we posited whether Zn^2+^ is critical for the inhibitory activity of AcrIIA28. Thus, we performed a functional assay for AcrIIA28 on the activity of SpyCas9 using the AcrIIA28 sample with Zn^2+^ removed. This sample was prepared by adding EDTA during the purification steps. EDTA used for chelating Zn^2+^ was finally removed by another SEC process. ICP-MS confirmed the absence of Zn^2+^ in the final AcrIIA28 sample. Although Zn^2+^ was not wholly removed, more than half of the Zn^2+^ was eliminated by adding EDTA (Figure 3D). We performed concentration-dependent inhibition assays on this sample (AcrIIA28 (EDTA)) and compared its activity with that of the sample without Zn^2+^ removal (AcrIIA28). The results showed that while the AcrIIA28 (EDTA) sample exhibited nearly identical activity to AcrIIA28 sample at lower concentrations, its ability to inhibit DNA cleavage activity of Cas9 significantly decreased at higher concentrations, around 1 μM. This finding suggests that Zn^2+^ indeed affects the activity of AcrIIA28, and particularly at higher concentrations, it plays a crucial role in effectively inhibiting Cas9 activity (Figure 3E).

### AcrIIA28 specifically binds to SpyCas9 via the REC3 domain

**Figure 4.**
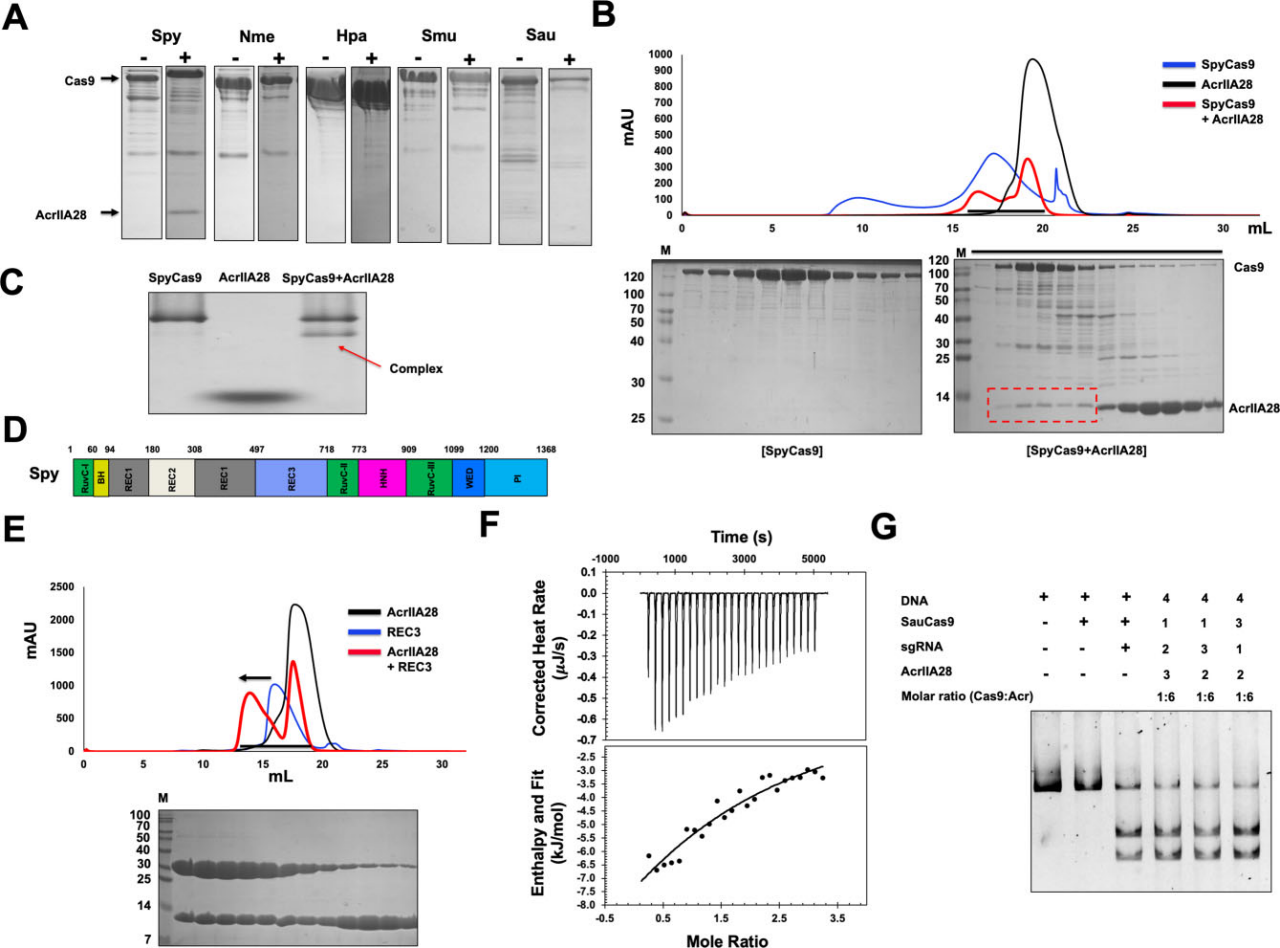
AcrIIA28 directly binds to only SpyCas9 via the REC3 domain. (**A**) Interaction analysis between AcrIIA28 and various Cas9s by SEC followed by SDS-PAGE analysis. SDS-PAGE gels are produced by loading one of the main fractions from the Cas9 sample with (+AcrIIA28) or without (-AcrIIA28) AcrIIA28. The black arrow indicates the co-migration of AcrIIA28 with Cas9. (**B**) Interaction analysis between AcrIIA28 and SpyCas9 by SEC followed by SDS-PAGE analysis. SEC profiles produced by AcrIIA28 (black line), SpyCas9 (blue line), and the mixture of AcrIIA28 and SpyCas9 (red line) are shown. SDS-PAGE gels produced by SpyCas9 alone and the mixture of AcrIIA28 and SpyCas9 are provided under the SEC profile. The red dot box on the SDS-PAGE gel indicates the co-migrated AcrIIA28 bands. Black lines indicate loaded fractions of SpyCas9/AcrIIA28 mixture for SDS-PAGE. M indicates protein size marker. (**C**) Native PAGE of SpyCas9, AcrIIA28 and SpyCas9 + AcrIIA28 mixture. The red arrow indicates a newly produced band that a complex might form. (**D**) Domain organization of SpyCas9. (**E**) Interaction analysis between AcrIIA28 and SpyREC3 domain by SEC. SEC profiles produced by AcrIIA28 (black line), SpyREC3 domain (blue line), and the mixture of AcrIIA28 and SpyREC3 domain (red line) are shown. SDS-PAGE gel produced by the AcrIIA28 and SpyREC3 domain mixture is provided under the SEC profile. Loaded fractions for SDS-PAGE were indicated by the black bar. The black arrow indicated peak movement on the SEC profile. (**F**) ITC experiment showing titration of AcrIIA28 into a SpyREC3 solution. The raw calorimetric titration data are shown in the upper panel, and experimental fitting of the data to a single site interaction model is shown in the lower panel. (**G**) An *in vitro* anti-CRISPR activity assay of AcrIIA28 against SauCas9. Polyacrylamide gels (4%) were stained with SYBR GOLD. The numbers indicate the order in which the agents were added to the reaction: one represents the sample added first, and four represents the sample added last. In the enzyme reaction, + and – indicate added and not added, respectively. All the experiments for figure 4 were performed three times with similar results.

Various strategies employed by Acrs to inhibit Cas9 have been proposed previously, including directly binding to the target DNA or sgRNA and to the Cas9 protein itself (29,30,45–50). Building upon this previous research, the inhibition mechanism of AcrIIA28 was further explored by performing a binding assay using the full-length Cas9 proteins from different species, such as *Streptococcus pyogenes* (SpyCas9), *Neisseria meningitidis* (NmeCas9), *Haemophilus parainfluenzae* (HpaCas9), *Simonsiella muelleri* (SmuCas9) and *Staphylococcus aureus* (SauCas9). When purified, AcrIIA28 was mixed with various Cas9 proteins, incubated, and loaded onto the SEC column; AcrIIA28 only co-migrated with SpyCas9, indicating that AcrIIA28 only specifically binds to SpyCas9 among the tested proteins (Figure 4A, B and Supplementary Figure S1). To confirm the interaction between AcrIIA28 and SpyCas9, we performed native PAGE. When AcrIIA28 was mixed with SpyCas9 and applied to native PAGE, the AcrIIA28 band completely disappeared, and a new band was produced immediately under the SpyCas9 band. This indicates that the newly formed band was produced by the AcrIIA28/SpyCas9 complex (Figure 4C).

For a more comprehensive understanding of the binding mechanism, further tests were conducted using individual SpyCas9 domains. As SpyCas9 is composed of distinct functional domains, including RuvC, REC, HNH, WED and PI, various expression constructs were generated to purify each domain for *in vitro* interaction tests (Figure 4D). Among the tested domains, we obtained soluble proteins for the REC1–2, REC3, HNH, WED domains of SpyCas9. The PI domain was insoluble in our experiments. We also employed the SEC binding tests to observe the interaction between AcrIIA28 and each SpyCas9 domain. According to these experiments, no obvious co-migration with AcrIIA28 was observed for the REC1–2, HNH and WED domains, indicating that AcrIIA28 does not interact with these domains (Supplementary Figure S2). However, obvious co-migration was observed with the REC3 domain. In addition, distinct peak movement was detected for the AcrIIA28/REC3 mixture sample (Figure 4E). These findings suggest that AcrIIA28 and the SpyCas9 REC3 domain bind directly. This direct interaction between AcrIIA28 and the REC3 domain was quantitatively analyzed using isothermal titration calorimetry (ITC). AcrIIA28 (1.2 mM) was titrated into 100 μM of the REC3 domain with 25 injections for incremental ITC experiments (Figure 4F). The released heat agreed with the expected values for an ideal interaction, indicating a single type of binding site without distinct cooperation in the interaction. Control titrations, performed without REC3, were measured and subtracted from the experimental titration curve. The resulting dissociation constants were 243 μM, indicating that AcrIIA28 directly interacts with SpyCas9 via the REC3 domain with low affinity. Native PAGE also confirmed the direct interaction between AcrIIA28 and the REC3 domains. When AcrIIA28 was mixed with REC3 and applied to native PAGE, the main REC3 peak on the native PAGE was completely absent, and a new band was produced just above the main band of AcrIIA28, indicating that the newly formed band was produced by the AcrIIA28/REC3 complex (Supplementary Figure S3). Overall, these collective findings from the binding assays involving SpyCas9 and its individual functional domains strongly suggest that the inhibitory effect of AcrIIA28 is achieved through a direct interaction with Cas9. Specifically, AcrIIA28 appears to target the REC3 domain of the SpyCas9 protein selectively.

Since AcrIIA28 can interact with only SpyCas9, we wondered if AcrIIA28 can inhibit other Cas9s that did not interact with AcrIIA28. To test this, SauCas9 was representatively selected and added to the inhibitory activity test alongside AcrIIA28. This test showed that the target DNA cleavage activity of SauCas9 was not inhibited by the addition of AcrIIA28, indicating that the direct interaction of AcrIIA28 to Cas9 is critical for the AcrIIA28 function (Figure 4G). In addition, we concluded that AcrIIA28 specifically inhibits the *S. pyrogen* Cas9.

### Biding mode analysis indicated that residues K7, W45 and S81 in AcrIIA28 are critical for Cas9 binding

**Figure 5.**
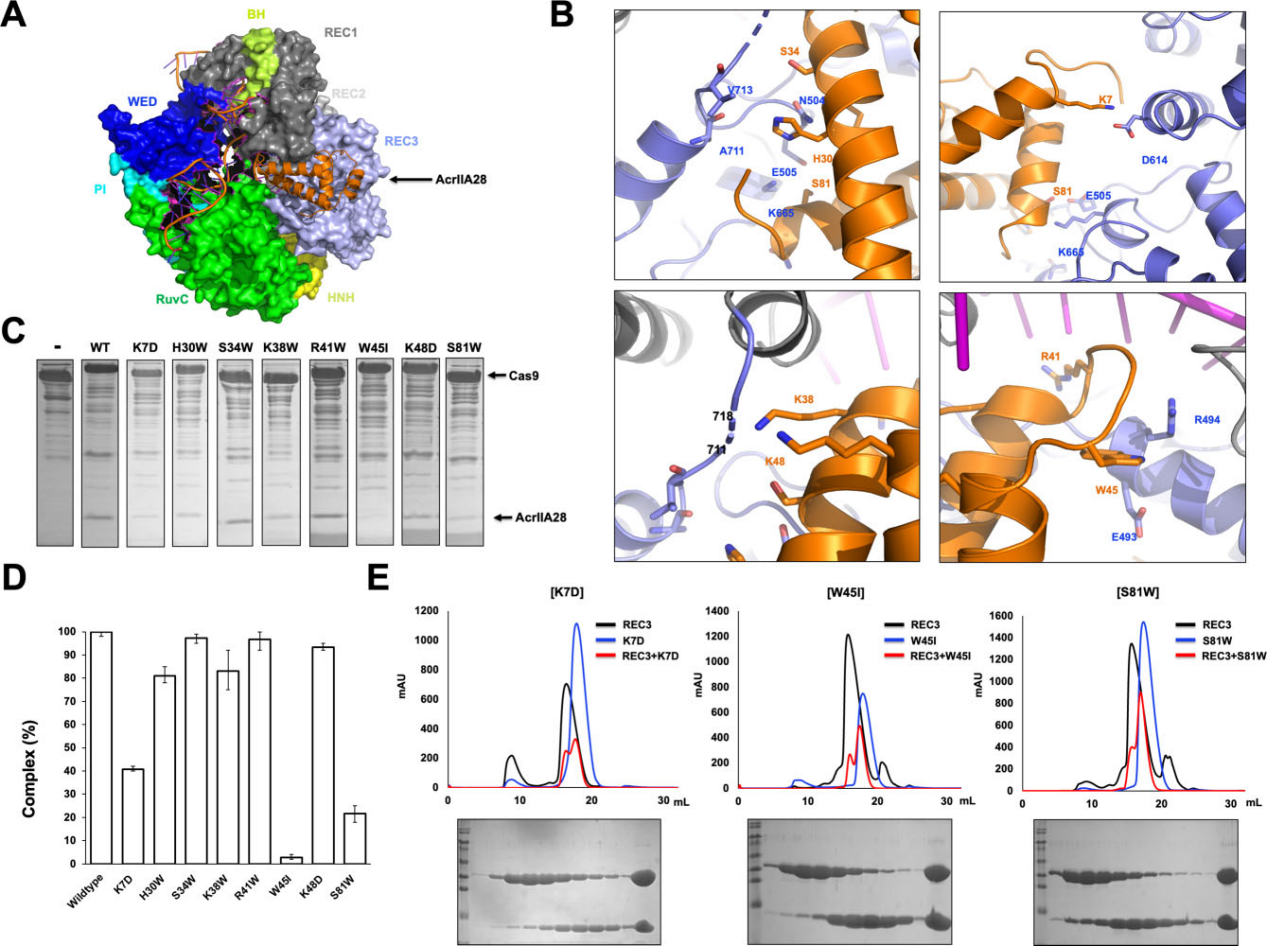
Biding mode analysis indicated that the AcrIIA28 residues K7, W45 and S81 are critical for AcrIIA28 binding to Cas9. (**A**) The docking model of AcrIIA28 docked onto SpyCas9. Cas9 and docked AcrIIA28 (orange color) are presented by surface and cartoon models, respectively. (**B**) Analysis of PPI detail from the docking model of AcrIIA28/SpyCas9 complex. Four PPI regions are independently magnified. The residues involved in the interaction are labeled. (**C**) Interaction analysis between SpyCas9 and various tentative PPI-disrupting mutants of AcrIIA28 by SEC followed by SDS-PAGE analysis. SDS-PAGE gels produced by loading one of the main fractions from the SpyCas9 sample with wild-type or various mutants are shown. A black arrow indicates acrIIA28 mutants that co-migrated with SpyCas9. (**D**) The bar chart shows the quantified intensity of the co-eluted AcrII28 mutants. Data are presented as the mean ± standard deviation from three independent experiments. (**E**) Interaction analysis between SpyREC3 and three PPI-disrupting mutants (K7D, W45I and S81W) of AcrIIA28 by SEC followed by SDS-PAGE analysis.

To gain insights into the interaction mechanisms between AcrIIA28 and SpyCas9 via the REC3 domain, docking analysis was conducted using the HDOCK server (42) to generate a complex AcrIIA28/Cas9 interaction model. This model suggested that AcrIIA28 was positioned in the REC3 domain of SpyCas9, which aligned with the results of the *in vitro* interaction analysis (Figure 5A). According to the docking model, we realized that residues K7, H30, S34, K38, K48, R41, W45 and S81 of AcrIIA28 might be essential for the interaction between AcrIIA28 and the REC3 domain in SpyCas9 (Figure 5B). Based on this analysis, all the analyzed residues were individually mutated to K7D, H30W, S34W, K38W, R41W, W45I, K48D and S81W and each mutant was purified (Supplementary Figure S4), to validate the docking complex model. Then, the resulting mutants were analyzed using the co-migration assay to determine their binding affinity to SpyCas9. Notably, the K7D, W45I and S81W mutants displayed significantly reduced binding to SpyCas9 compared to the wild-type AcrIIA28 (Figure 5C and Supplementary Figure S5). The bar graph depicting the pull-down tendencies of AcrIIA28 and its mutants further corroborated this finding, confirming the weakened binding of the K7D, K48D and S81W mutants to SpyCas9 (Figure 5D).

The effect of the protein–protein interface (PPI) disrupted mutants was confirmed by performing interaction tests on various PPI-disrupting mutants of AcrIIA28 with the REC3 domain of SpyCas9. This analysis also showed that K7D, W45I, and S81W did not co-migrate with the REC3 domain based on the SEC analysis (Figure 5E). In contrast, the last mutants still co-migrated with the REC3 domain (Supplementary Figure S6), indicating that the docking model might be accurately predicted and that the PPI residues K7, W45 and S81 of AcrIIA28 are critical in recognizing the REC3 domain of SpyCas9.

### Inhibition of Cas9 by AcrIIA28 is significantly dependent on the direct interaction

**Figure 6.**
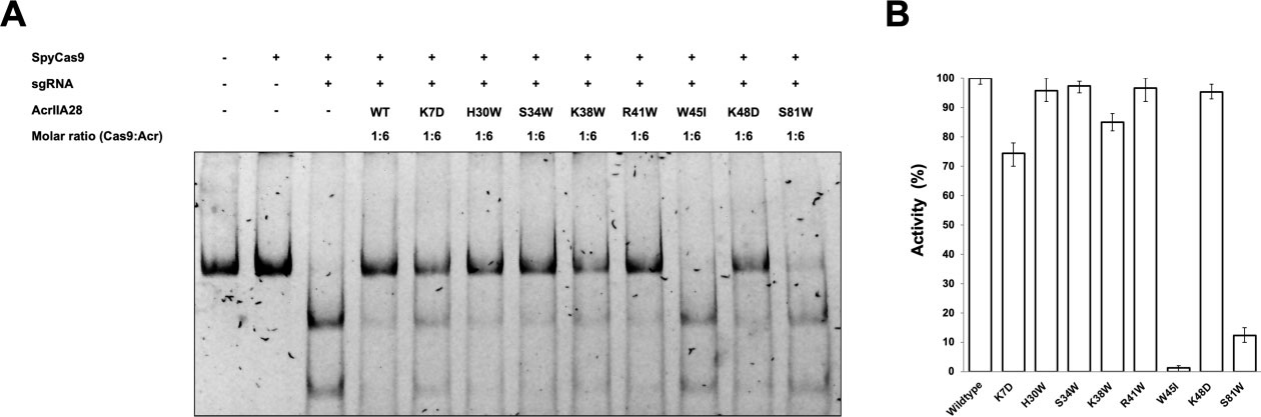
Direct interaction of AcrIIA28 is critical for Cas9 inhibition. (**A**) *In vitro* target DNA cleavage assay using wild-type Cas9 and various AcrIIA28 mutants. *In vitro* anti-CRISPR activity assay using various tentative PPI-disrupting mutants of AcrIIA28. In the enzyme reaction, + and – indicate added and not added, respectively. (**B**) Quantitative histogram of anti-Cas9 activity of AcrIIA28 according to (A). The inhibitory activity of wild-type AcrIIA28 was considered to be 100%. Data are presented as the mean ± standard deviation from three independent experiments.

The postulated mechanism through which AcrIIA28 is inhibited involves its direct interaction with the REC domain of Cas9. This hypothesized mechanism was verified using an assay that evaluates the cleavage/inhibition activity, employing various AcrIIA28 mutants. The assay was conducted under standardized conditions, differing solely in the AcrIIA28 variant being utilized. In comparison to the wild-type AcrIIA28, which effectively hindered target cleavage activity of Cas9, the mutants K7D, W45I and S81W exhibited more pronounced cleaved bands, signifying a reduction in Cas9 inhibition (Figure 6A and B). Among the three mutants, W45I had the most dramatic effect, indicating that W45 is the critical residue for recognizing Cas9 and the inhibitory activity of AcrIIA28. These observations collectively provide robust substantiation for the envisioned inhibitory mechanism of AcrIIA28 by underscoring that the confluence of direct binding assumes a pivotal role in the capability of AcrIIA28 to counteract the target DNA cleavage activity of Cas9.

### The proposed scenario of SpyCas9 inhibition by AcrIIA28

**Figure 7.**
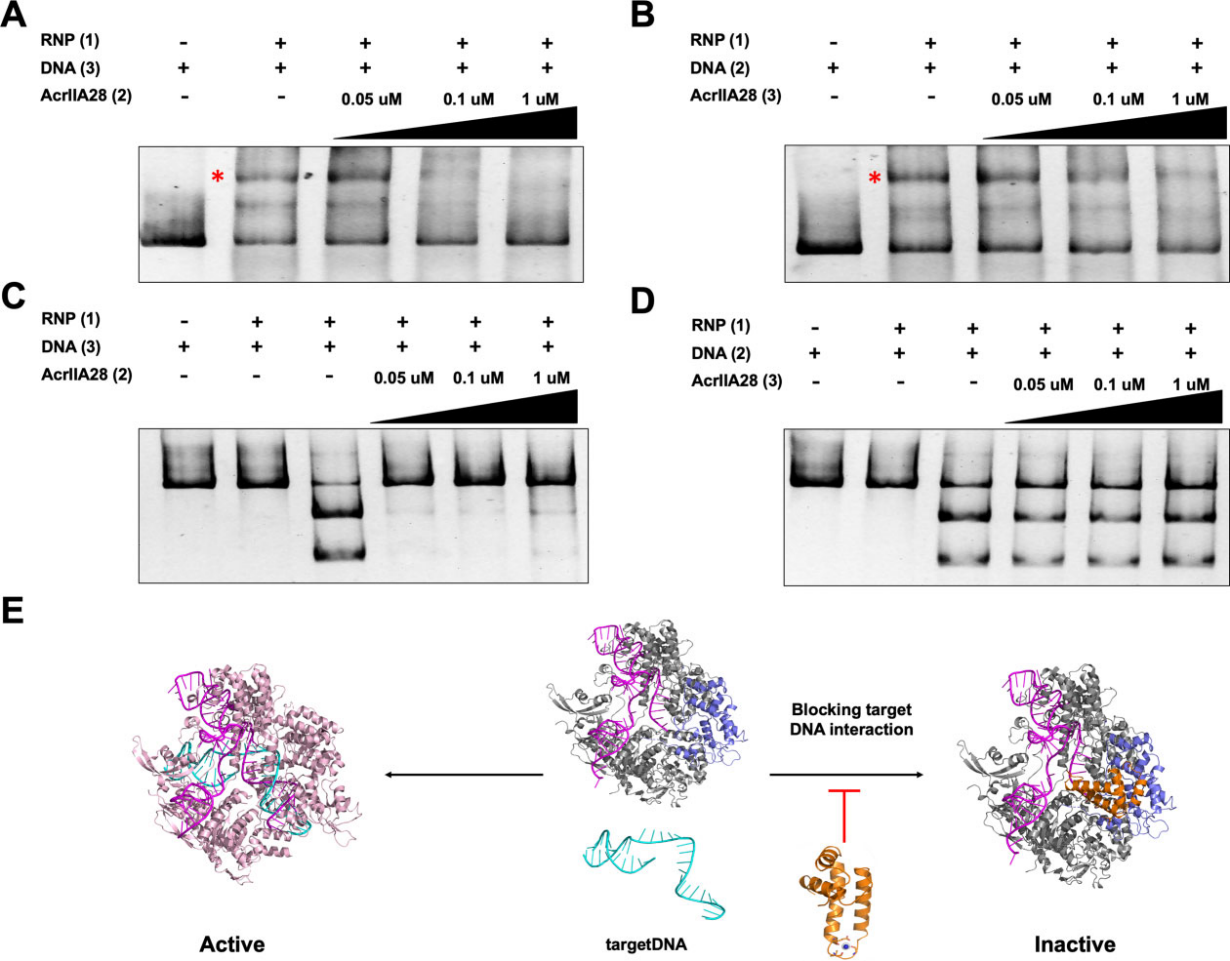
Proposed model of Cas9 inhibition by AcrIIA28. (**A** and **B**) EMSA analysis of the effect of AcrIIA28 on the binding of target DNA into Cas9. The number next to each protein indicates the order in which the proteins were added to form the complex. The red star indicates the band newly produced by the RNP/DNA complex. The experiments were performed three times with similar results. (**C** and **D**) An *in vitro* anti-CRISPR activity assay was used to compare the inhibitory effect of AcrIIA28 when added before (C) or after (D) the target DNA. Polyacrylamide gels (4%) were stained with SYBR GOLD. The amount of AcrIIA28 added to the reaction is indicated. The numbers next to the agents used in the experiment indicate the order in which agents were added to the reaction: one represents the sample added first, and three represents the sample added last. In the enzyme reaction, + and – indicate added and not added, respectively. (**E**) Proposed model of Cas9 inhibition by AcrIIA28.

Presently, more than 100 distinct anti-CRISPR (Acr) proteins that inhibit various CRISPR–Cas systems, including type I, II, III, V and VI, have been identified. Notably, 32 AcrIIA families have been discovered using diverse methodologies such as ‘self-targeting’, ‘machine learning’ and others (27,31,51,52). AcrIIA proteins exhibit a range of inhibitory mechanisms against Cas9, which can be categorized into three main strategies: Interference with crRNA loading, prevention of DNA binding, and blockage of DNA cleavage (29,53). To elucidate the detailed inhibitory mode of action of AcrIIA28 against SpyCas9, we used EMSA analysis to determine whether direct binding of AcrIIA28 to SpyCas9 inhibits the target DNA recruitment into SpyCas9, which is one of the most common ways for the AcrIIA family to prevent Cas9 activity. Once the RNP complex was incubated with the target DNA, target DNA was detected at the upper position by producing a new band that might be produced by the RNP/DNA complex (Figure 7A). However, when AcrIIA28 was added to the RNP complex prior to the addition of the target DNA, the target DNA failed to produce an RNP/DNA complex band, thereby indicating that when AcrIIA28 bound to the RNP complex, it blocked the target DNA from binding to SpyCas9 (Figure 7A). When target DNA was added to the RNP complex prior to the addition of AcrIIA28, the RNP/DNA complex was still produced (Figure 7B). This indicated that AcrIIA28 blocks the target DNA from being loaded onto Cas9 and once the RNP/DNA complex was performed, AcrIIA28 could not force the DNA to separate from Cas9. To confirm our insist, we conducted *in vitro* cleavage assays, comparing the inhibitory effect of AcrIIA28 when added before or after the target DNA. This experiment showed that the inhibitory activity of AcrIIA28 was observed when added before the target DNA, whereas its activity was nullified when added after the target DNA (Figure 7C and D). This outcome unequivocally validates our assertion that AcrIIA28 impedes target DNA binding on Cas9.

Finally, based on our observations, we concluded that AcrIIA28 can inhibit the DNA-cleavage activity of SpyCas9 by directly interacting with the REC3 domain, which then can prevent the target DNA from being loaded into the Cas9 active site (Figure 7E).

## Discussion

The identification of AcrIIA28 from *Streptococcus phage Javan128* and its function as a type IIA anti-CRISPR protein, which potently inhibits the Cas9, was suggested by Song and colleagues in 2022 (33). However, they showed that the possible function of Acr in AcrIIA28, the molecular mechanism of AcrIIA28, remains unknown. This study explored the tentative inhibitory mechanism of AcrIIA28 against Cas9. We solved the crystal structure of AcrIIA28, composed of four α-helices with a novel fold, which had currently not been reported. AcrIIA28 directly interacted with only SpyCas9 and specifically inhibited the SpyCas9 activity. Within the AcrII family, there are instances where only specific species of Cas9 are inhibited by certain Acr proteins (such as AcrIIA4, AcrIIA6, and AcrIIC3) (30,45,46,54). In contrast, in other cases, various Cas9 proteins from different bacterial species are targeted (including AcrIIA5, AcrIIA11, AcrIIC1, AcrIIC4 and AcrIIC5) (45,50,55). The AcrIIA28 used in this study belongs to the former case. The SpyCas9 is one of the most widely used Cas effectors in genome engineering, particularly in the context of CRISPR technology (11,29). SpyCas9 is renowned for its versatility, efficiency, and specificity in targeting and modifying specific DNA sequences. Researchers have developed various versions of SpyCas9 with enhanced properties, such as increased specificity, altered PAM requirements, or reduced off-target effects, to refine further and expand its applications in genome engineering.

The mechanisms employed by Acrs to inhibit Cas9 have been extensively studied and revealed that Acrs utilize diverse strategies to disrupt Cas9 function, including (i) directly binding to target DNA or sgRNA, (ii) blocking Cas9 from binding to target DNA by directly interacting with Cas9 and (iii) masking the Cas9 cleavage domains by directly interacting with Cas9 (30,45,46,50,54,55). Our investigation found that AcrIIA28 directly binds to only SpyCas9 proteins, explicitly targeting the REC3 domain. Although several type II Cas9 inhibitors, including AcrIIA4, AcrIIA2, AcrIIC4 and AcrIIC5, have been identified to prevent Cas9 cleavage by directly binding to target Cas9, targeting the REC3 domain employed by our AcrIIA28, was the first case. Despite differences in their protein structures, AcrIIA4 and AcrIIA2 share a common feature of interacting with the PI domain in Cas9, thereby obstructing the PAM region (30,49). This PAM-blocking strategy was the most popular in the AcrIIA family, which can directly bind to Cas9. The REC3 binding Acr is the first reported in the AcrIIA28 family.

After Acr binds to Cas9, most Acrs block the target DNA from being loaded into the Cas9 active site. Several type II Cas9 inhibitors, including AcrIIA4, AcrIIA2, AcrIIC4 and AcrIIC5, have been identified to prevent Cas9 cleavage by inhibiting target DNA binding. AcrIIA28 also uses the same inhibitory strategy to block the loading of target DNA by directly interacting with Cas9 via the REC3 domain. The binding affinity of AcrIIA28 to REC3 was rather modest and we thought that AcrIIA28 may not require very strong binding to exert its effects. Our rationale for this is based on the structural modeling, which revealed that AcrIIA28 forms a very small protein-protein interaction (PPI) interface between the REC3, REC1 and RUVC domains. Despite this small interface, AcrIIA28 exhibits a remarkable ability to specifically inhibit SpyCas9 activity. It is often the case that very small binding interfaces do not result in very strong binding. Considering that Cas9 undergoes dynamic structural changes during its activation, we hypothesized that AcrIIA28 may have been designed to bind to these small regions to achieve its function effectively, even in the presence of structural changes in Cas9. Therefore, it seems that the small binding interface of AcrIIA28 may be intentional to allow it to effectively interact with Cas9, which can modify their structure during activation.

The meaning of the bound Zn^2+^ metal is still enigmatic. AcrIIA28 is the first Acr that contains the metal in the well-defined binding site. Our study showed that this metal affected the activity of AcrIIA28 at the high concentration. Based on this result, we concluded that proper Zn^2+^ binding was crucial for the effective inhibition of Cas9 activity.

An in-depth exploration of the inhibitory mechanism of Acrs against Cas9 serves a dual purpose: Enhancing comprehension of bacterial defense mechanisms and refining the precision and efficacy of CRISPR–Cas9 technology for genome editing. A comprehensive elucidation of the molecular underpinnings governing the inhibition of Ca9 by AcrIIA28 holds promise for developing strategies to augment the control and specificity of CRISPR-mediated gene editing endeavors.

## Supplementary Material

gkae357_Supplemental_File

## Data Availability

The coordinate and structural factors have been deposited into the Research Collaboratory for Structural Bioinformatics (RCSB) Protein Data Bank (PDB) under the PDB code 8K4M.
