# Supplementary material for: AcrIIA28 is a metalloprotein that specifically inhibits targeted-DNA loading to SpyCas9 by binding to the REC3 domain
Source: Nucleic Acids Res. 2024 May 10;52(11):6459–71. doi: 10.1093/nar/gkae357 (PMC11194106; doi:10.1093/nar/gkae357)
Supplement: gkae357_Supplemental_File [file gkae357_supplemental_file.pdf]

AcrIIA28 is a metalloprotein that specifically inhibits targeted-DNA loading to SpyCas9 by binding to the REC3 domain

Gi Eob Kim[1,2,], and Hyun Ho Park[1,2,*]

[1]College of Pharmacy, Chung-Ang University, Seoul 06974, Republic of Korea

[2]Department of Global Innovative Drugs, Graduate School of Chung-Ang University, Seoul 06974, Republic of Korea

*Correspondence to:
Hyun Ho Park; College of Pharmacy, Chung-Ang University, Seoul 06974, Republic of Korea;
Tel: +82-2-820-5930; Fax: +82-2-820-3033; Email: xrayleox@cau.ac.kr

Keywords:
anti-CRISPR; AcrIIA28; adaptive immunity; CRISPR–Cas system; crystal structure
structure

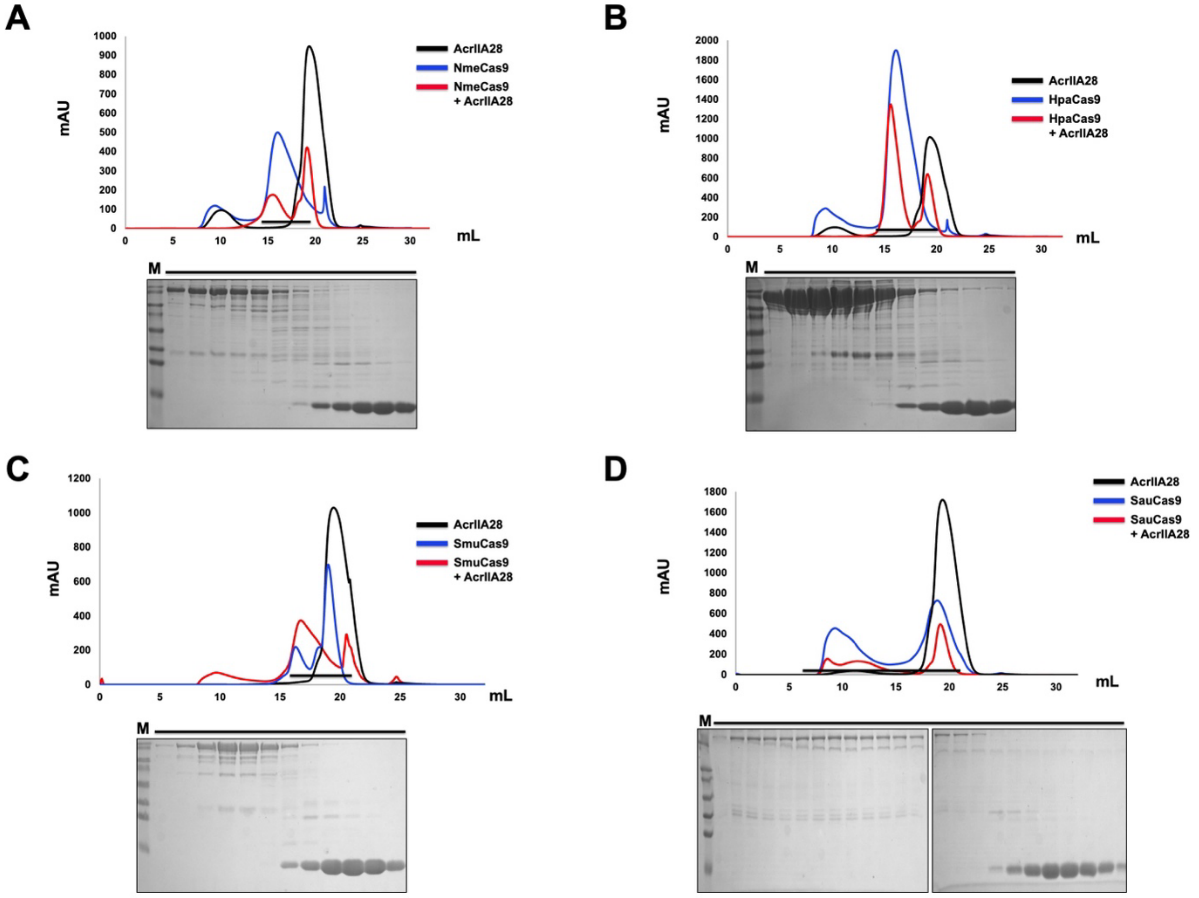

Supplementary Figure 1. Analysis of the interactions between AcrIIA28 and various different Cas9 from different species on SEC followed by SDS-PAGE. Purified AcrIIA28 was mixed with NmeCas9 **(A),** HpaCas9 **(B)**, SmuCas9 **(C)**, and SauCas9 **(D)** and loaded onto SEC for analyzing the co-migration tendency on SDS-PAGE. Loaded fractions are indicated by the horizontal black bar. The corresponding fractions from SEC loaded onto SDS-PAGE are also indicated by black bar.

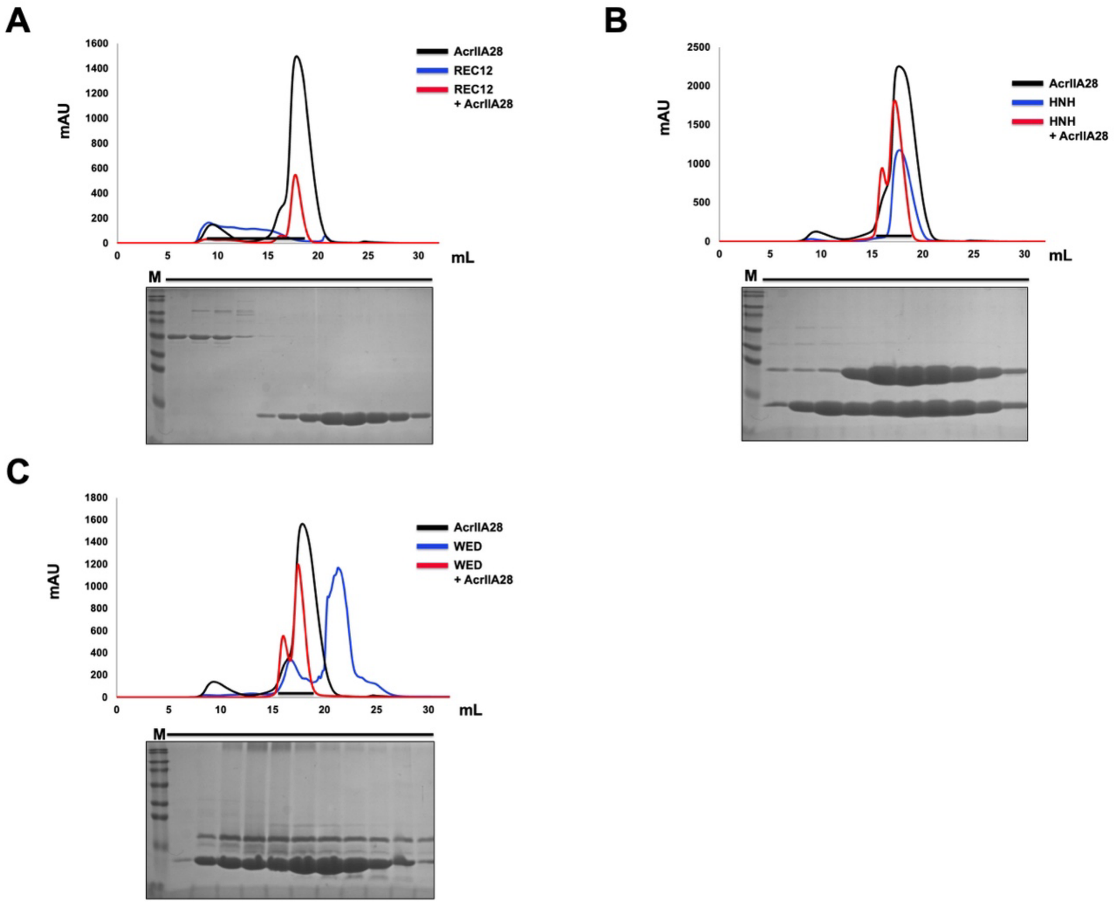

Supplementary Figure 2. Analysis of the interactions between AcrIIA28 and each domain of SpyCas9 on SEC followed by SDS-PAGE. AcrIIA28 was mixed with SpyREC12 **(A)**, SpyHNH **(B),** and SpyWED **(C)** and loaded onto SEC for analyzing the co-migration tendency on SDS-PAGE. Loaded fractions are indicated by the horizontal black bar. The corresponding fractions from SEC loaded onto SDS-PAGE are also indicated by black bar.

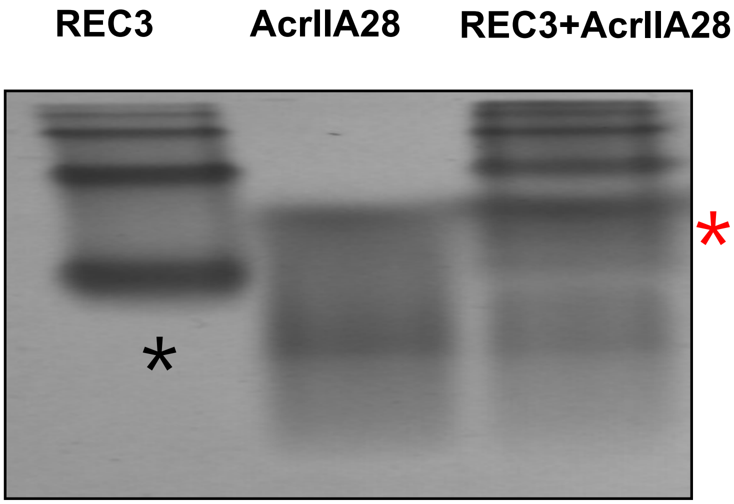

Supplementary Figure 3. Native PAGE of REC3, AcrIIA28, and REC3 + AcrIIA28 mixture. The black and red stars indicated the disappeared and a newly produced band, respectively.

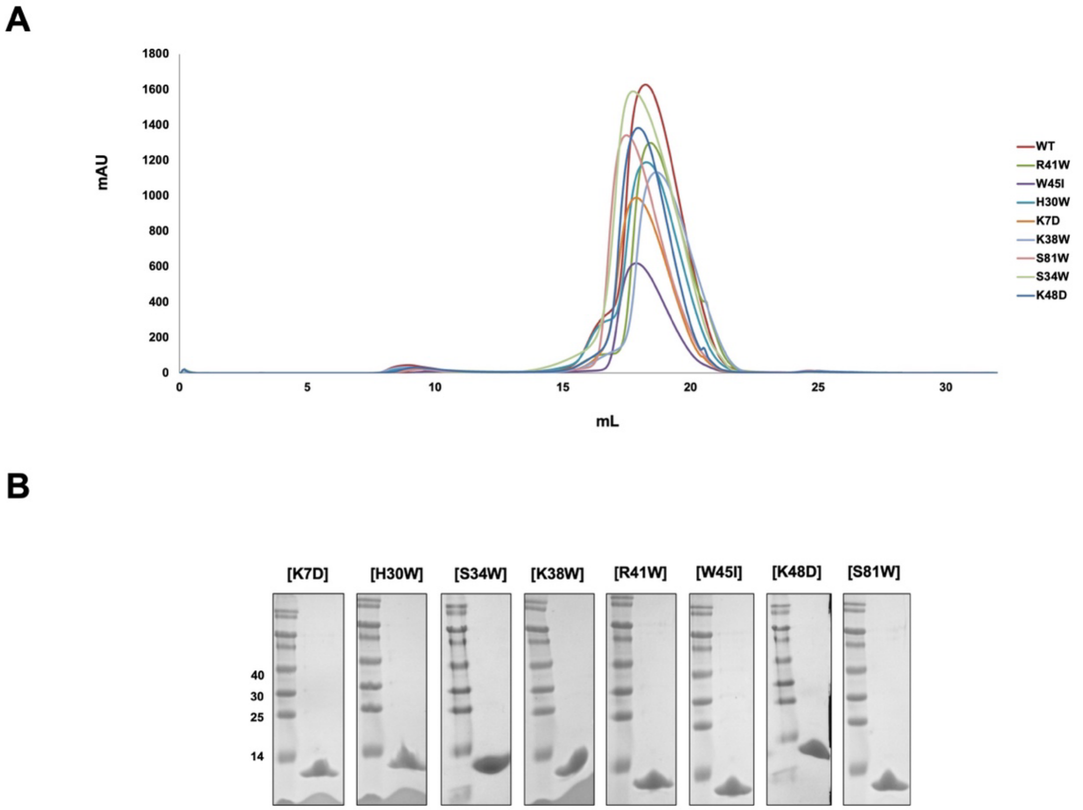

Supplementary Figure 4. Production of various mutants of AcrIIA28 used for interaction and activity analysis. (A) Purification of various mutatnts of AcrIIA28 by SEC. SEC profiles produced by each mutant were shown. **(B)** SDS-PAGE gel produced by finally concentrated samples of mutants of AcrIIA28.

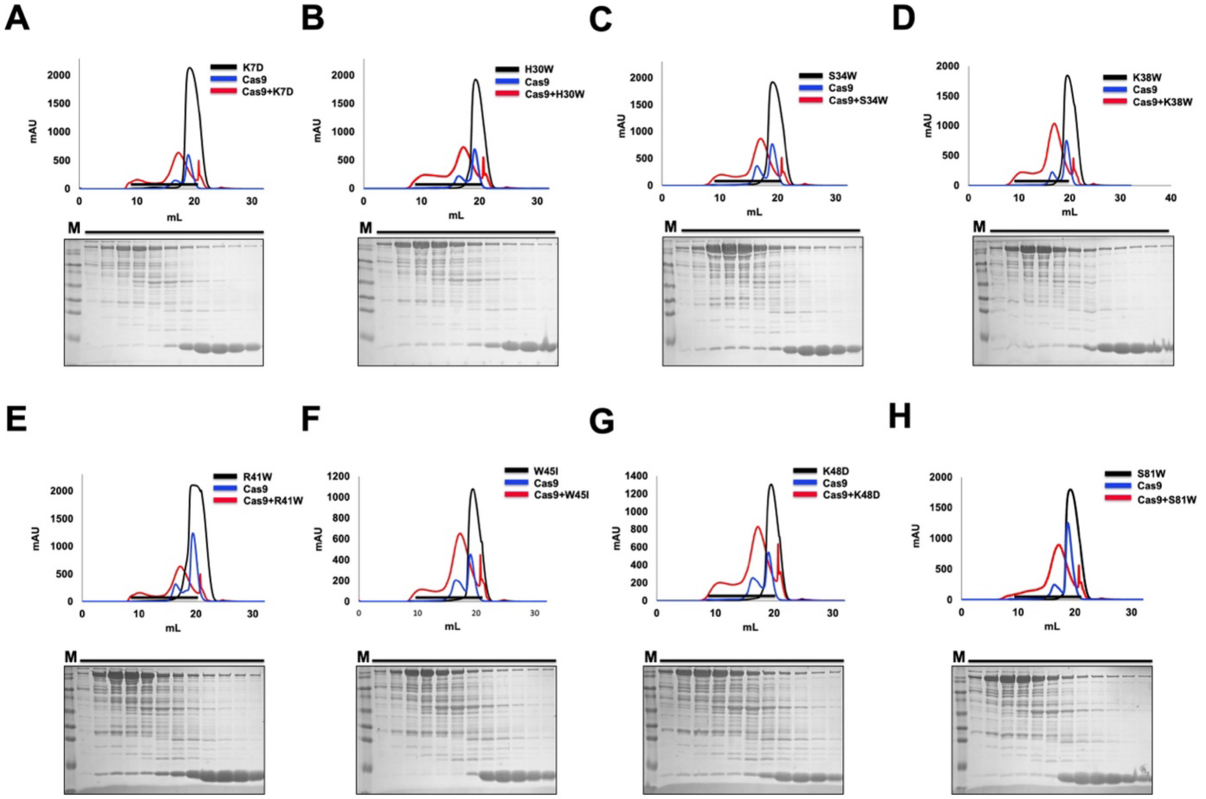

Supplementary Figure 5. Analysis of the interactions between SpyCas9 and various mutants of AcrIIA28 on SEC followed by SDS-PAGE. (A-H) Interaction analysis of SpyCas9 with various mutants of AcrIIA28 by SEC. SEC profiles produced by the mixture were shown. SDS-PAGE gel produced by main peak and excess AcrIIA28 mutants' peak fractions from the mixture was provided under the SEC profile. Loaded fractions are indicated by the horizontal black bar. The corresponding fractions from SEC loaded onto SDS-PAGE are also indicated by black bar.

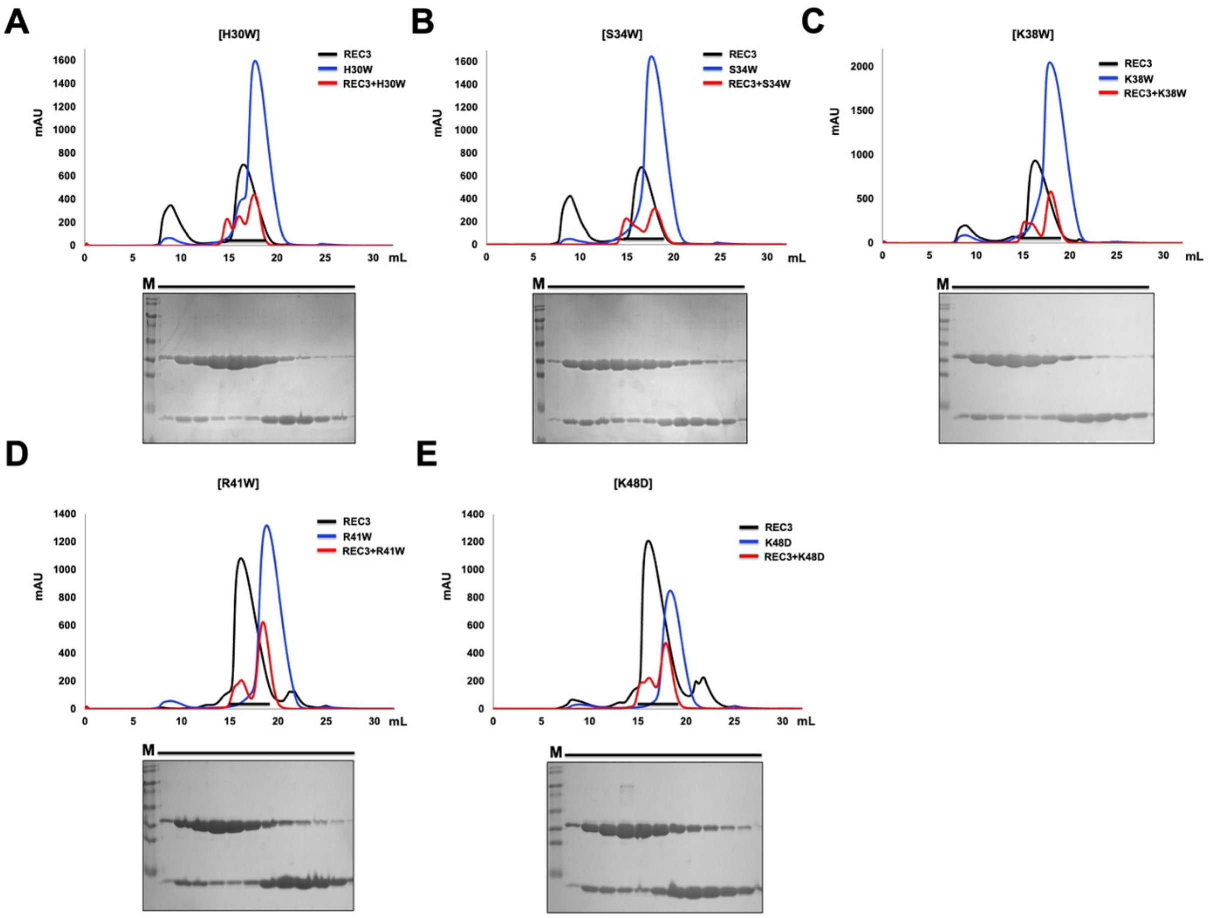

Supplementary Figure 6. Analysis of the interactions between SpyREC3 and various mutants of AcrIIA28 on SEC followed by SDS-PAGE. Interaction analysis of SpyCas9 with H30W **(A)**, S34W **(B)**, K38W **(C)**, R41W **(D)**, and K48D **(E)** by SEC. SEC profiles produced by the mixture were shown. SDS-PAGE gel produced by main peak and excess AcrIIA28 mutants' peak fractions from the mixture was provided under the SEC profile. Loaded fractions are indicated by the horizontal black bar. The corresponding fractions from SEC loaded onto SDS-PAGE are also indicated by black bar.

Supplementary Table1. Oligonucleotides used in this study.

Name	Sequence (5'→3')
AcrIIA28-F	GGGCATATGATGAAAACCATTTTTACCAA
AcrIIA28-R	GGGCTCGAGGCTATTATACAGAAATTTAT
K7D(Mut)- F	ACCATTTTTACCGATAAACAGACCGAAGAACT
K7D(Mut)- R	TTCTTCGGTCTGTTTATCGGTAAAAATGGTTTTCATCA
H30W(Mut)- sense	TTTAATAGCATGTGGGATTTTCGTAGTCAGCATGCAA
H30W(Mut)- antisense	CTGACTACGAAAATCCCACATGCTATTAAACAGTTCT
S34W(Mut)- sense	TGATTTTCGTTGGCAGCATGCAAAAGA
S34W(Mut)- antisense	CATGCTGCCAACGAAAATCATGCATGCT
K38W(Mut)- sense	TCAGCAYGCATGGGAAGCCCGTATTCC
K38W(Mut)- antisense	TACGGGCTTCCCATGCATGCTGACTAC
R41W(Mut)- sense	AAGAAGCCTGGATTCCGGGTTGGA
R41W(Mut)- antisense	AACCCGGAATCCAGGCTTCTTTTGCATGCT
W45I(Mut)-sense	CCGTATTCCGGGTATTAGCGATAAATATAATAAACTGGA
W45I(Mut)-antisense	ATTTATCGCTAATACCCGGAATACGGGCTT
K48D(Mut)- sense	TTGGAGCGATGATTATTATTAACTGGAAAAGAAAAT
K48D(Mut)- antisense	TCCAGTTTATTATAATCATCGCTCCAACCCGGAATA
K48W(Mut)- sense	CCGGGTTGGAGCGATTGGTATAATAAACTGGAA
K48W(Mut)- antisense	TTCCAGTTTATTATACCAATCGCTCCAACCCGG
K51W(Mut)- sense	GGAGCGATAAATATAATTGGCTGGAAAAGAAAATGCT
K51W(Mut)- antisense	AGCATTTTCTTTTCCAGCCAATTATATTTATCGCTCC
K55W(Mut)- sense	TAATAAACTGGAAAAGTGGATGCTGAGCGATTTTG
K55W(Mut)- antisense	CAAAATCGCTCAGCATCCACTTTTCCAGTTTATTA
D59W(Mut)- sense	GAAAATGCTGAGCTGGTTTGAAGAAGTTA
D59W(Mut)- antisense	TAACTTCTTCAAACCAGCTCAGCATTTTC
S81W(Mut)- sense	TGGGATAATCTGTGGAATAAATTTCTGTATAATAGCCT
S81W(Mut)- antisense	AGAAATTTATTCCACAGATTATCCCAAATCAGTTCGCT
SpyCas9_REC12-F	GGGCATATGGATGACTCATTTTTCCACCG
SpyCas9_REC12-R	GGGCTCGAGATTGGTCATACGTTCGATAA
SpyCas9_REC3-F	GGGCATATGTTCGATAAAAACCTGCCGAA
SpyCas9_REC3-R	GGGCTCGAGATCGCCTTGACCTGACACTT
SpyCas9_HNH-F	GGGCATATGGGCCAGAAAAACTCGCGTGAA
SpyCas9_HNH-R	GGGCTCGAGGGACAGACCGCCGCGTTCCG
SpyCas9_WED-F	GGGCATATGGAAGTGCAGACGGGCGGTTT
SpyCas9_WED-R	GGGCTCGAGTTTCGGCAGTTTGATGATCA
target DNA(mIRGM3)	ATGGCGAACTACCTGAAACGTCTGATCAGCCCGTGGTCTAAATCTATGACC<br>GCGGGTGAATCTCTGTACAGCAGCCAGAACTCTTCTAGCCCGGAAGTTATT<br>GAAGATATCGGTAAAGCGGTTACCGAAGGCAATCTGCAGAAAGTTATCGG<br>TATCGTTAAAGATGAAATTCAGTCTAAATCTCGTTACCGTGTGAAAATTGC<br>GGTTACCGGCGATTCTGGTAACGGCATGAGCTCCTTCATCAACGCACTGCG<br>TTTCATCGGTCACGAAGAAGAAGATTCTGCGCCGACCGGTGTTGTTCGTAC<br>CACCAAAAAACCGGCATGCTACAGCTCTGATAGCCACTTCCCGTACGTTGA<br>ACTGTGGGACCTGCCGGGTCTGGGCGCTACCGCTCAGTCTGTTGAATCTTA<br>CCTGGAAGAAATGCAGATTAGCACCTTCGATCTGATCATTATCGTTGCGTC<br>TGAACAGTTTAGCTCTAACCACGTTAAACTGGCGATCACCATGCAGCGTAT<br>GCGTAAACGTTTCTATGTTGTTTGGACTAAACTGGATCGCGATCTGTCTACT<br>TCTACTTTCCCGGAACCGCAGCTGCTGCAGTCCATCCAGCGTAACATTCGC<br>GAAAACCTGCAGCAGGCTCAGGTTCGTGACCCGCCGCTGTTCCTGATCAGC<br>TGCTTCAGCCCGTCCTTCCACGATTTCCCGGAACTGCGTAACACCCTGCAG<br>AAAGACATCTTCAGCATCCGTTATCGTGATCCGCTGGAAATCATCTCTCAG<br>GTTTGTGATAAATGCATTAGCAACAAAGCCTTCAGCCTGAAAGAAGATCAG

	ATGCTGATGAAAGATCTGGAAGCAGCAGTTAGCTCTGAAGATGATACCGC GAACCTGGAACGTGGCCTGCAGACCTATCAGAAACTGTTTGGTGTTGATGA TGGTTCTCTGCAGCAGGTTGCGCGTTCTACCGGTCGTCTGGAAATGGGCAG CCGTGCGCTGCAGTTCCAGGACCTGATTAAAATGGATCGTCGTCTGGAACT GATGATGTGCTTCGCGGTTAACAAATTCCTGCGCCTGCTGGAAAGCTCTTG GTGGTATGGCCTGTGGAACGTTGTTACCCGTTACTTCCGTCACCAGCGTCAC AAACTGGTTATCGAAATCGTTGCGGAAAACACCAAAACCAGCCTGCGTAA AGCGCTGAAAGATA
Target oligo- sense (for sgRNA synthesis)	TTCTAATACGACTCACTATAGCAGCTGATCAGGAACAGCGGTTTTAGTACT CTGGAAACAGAATCTACTAAAACAAGGCAAAATGCCGTGTTTATCTCGTCA ACTTGTTGGCGAGATTT
Target oligo- antisense (for sgRNA synthesis)	AAATCTCGCCAACAAGTTGACGAGATAAACACGGCATTTTGCCTTGTTTTA GTAGATTCTGTTTCCAGAGTACTAAAACCGCTGTTCCTGATCAGCTGCTATA GTGAGTCGTATTAGAA
SpyCas9 sgRNA	AACGAUAAUGAUCAGAUCGAGUUUUAGAGCUAGAAAUAGCAAGUUAAA AUAAGGCUAGUCCGUUAUCAACUUGAAAAAGUGGCACCGAGUCGGUGCU UUU
SauCas9 sgRNA	GCAGCUGAUCAGGAACAGCGGUUUUAGUACUCUGGAAACAGAAUCUACU AAAACAAGGCAAAAUGCCGUGUUUAUCUCGUCAACUUGUUGGCGAGAUU U
